# *Staphylococcus caledonicus* sp. nov. and *Staphylococcus canis* sp. nov. isolated from healthy domestic dogs

**DOI:** 10.1099/ijsem.0.004878

**Published:** 2021-07-23

**Authors:** Logan L. Newstead, J. Harris, S. Goodbrand, K. Varjonen, T. Nuttall, Gavin K. Paterson

**Affiliations:** ^1^​ Royal (Dick) School of Veterinary Studies and The Roslin Institute, University of Edinburgh, Easter Bush Campus, Midlothian EH25 9RG, UK; ^2^​ AniCura Djursjukhuset Albano, Rinkebyvägen 21A, 182 36 Danderyd, Sweden

**Keywords:** novel taxa, *Staphylococcus*, veterinary microbiology

## Abstract

Two strains, H8/1^T^ and H16/1A^T^, of Gram-stain-positive, coagulase-negative staphylococci were isolated from separate healthy domestic dogs in Scotland. Both strains were genome sequenced and their inferred DNA–DNA hybridisation indicates that H8/1^T^ and H16/1A^T^ represent two novel species of the genus *Staphylococcus*. On the basis of the results of genome sequence analysis (genome blast distance phylogeny and single nucleotide polymorphism analysis) H8/1^T^ is most closely related to *Staphylococcus devriesei* and H16/1A^T^ most closely related to *Staphylococcus felis*. Also, average nucleotide identity distinguished H8/1^T^ and H16/1A^T^ from *S. devriesei* and *S. felis* as did minor phenotypic differences. On the basis of these results, it is proposed that H8/1^T^ and H16/1A^T^ represent novel species with the respective names *Staphylococcus caledonicus* and *Staphylococcus canis*. The type strain of *S. caledonicus* is H8/1^T^ (=NCTC 14452^T^=CCUG 74789^T^). The type strain of *S. canis* is H16/1A^T^ (=NCTC 14451^T^=CCUG 74790^T^)

At the time of writing the genus *Staphylococcus* [1, 2] of Gram-stain-positive, coccus-shaped bacteria consists of 55 species (https://lpsn.dsmz.de/genus/staphylococcus, accessed 20th March 2020) [3]. Typically, staphylococci are found as commensal inhabitants of the skin and mucous membranes in a wide range of animal hosts, particularly in humans, other mammals and birds. The genus includes several important human and veterinary opportunistic pathogens, such as *Staphylococcus aureus*, *Staphylococcus epidermidis*, *Staphylococcus lugdunensis* and *Staphylococcus pseudintermedius*. Two canine staphyloccocal strains H8/1^T^ and H16/1A^T^ have been characterised, for which the respective names of *Staphylococcus caledonicus* sp. nov. and *Staphylococcus canis* sp. nov. are proposed.

*S. caledonicus* H8/1^T^ and *S. canis* H16/1A^T^ were isolated in 2018 from multisite swabs (a single swab sampling the nares, axilla, groin and perineum) from separate healthy adult dogs at the Royal (Dick) School of Veterinary Studies, University of Edinburgh, Scotland, UK. These samples were collected as part of an epidemiological study to isolate and genome sequence canine commensal bacteria. The dogs had no clinical signs or history of skin disease and had not received antimicrobial treatment for at least 12 months prior to sampling. Swabs were cultured initially by salt broth enrichment, statically at 37 °C for 24 h [tryptone soya broth (Oxoid) plus 6.5 % w/v sodium chloride] before plating onto mannitol salt agar (Oxoid) and then incubation at 37 °C for 24 h. Both isolates are Gram-stain-positive, catalase-positive cocci and were whole-genome sequenced using HiSeq technology (Illumina) with 2×250 bp paired-end reads, read trimming and assembly (performed by Microbes NG, Birmingham, UK). Reads were trimmed using Trimmomatic version 0.30 [4], using a sliding window quality cut-off of 15. Genome assembly was done *de novo* using SPAdes version 3.7 [5], with default parameters for 250 bp Illumina reads. Assemblies were annotated using the NCBI Prokaryotic Genome Annotation Pipeline [6].

To identify these two strains to the species level their genome sequences were uploaded onto the Type (Strain) Genome Server (TYGS) (https://tygs.dsmz.de/) [7]. The results of this genome-based analysis support the proposal of the two strains as each representing a novel species of the genus *Staphylococcus*. The TYGS phylogenetic tree generated by the Genome blast Distance Phylogeny approach (GBDP) indicated that H8/1^T^ is most closely related to, but distinct from *Staphylococcus devriesei* CCUG 58238^T^ with H16/1A^T^ most closely related to, but distinct from *Staphylococcus felis* DSM 7377^T^ (Fig. 1). A 16S rRNA gene phylogeny, produced by TYGS, also indicated that H8/1^T^ is most closely related to *S. devriesei* and H16/1A^T^ most closely related *S. felis* (Fig. 2). Although, 16S rRNA gene sequences often lack sufficient variation to differentiate between species of the genus *Staphylococcus* in pairwise comparisons [8–11]. Indeed, neither H8/1^T^ nor H16/1A^T^ could be differentiated from *S. devriesei* or *S. felis* type strains respectively on the basis of 16 rRNA gene sequence, Table 1. Nonetheless, designation of both H8/1^T^ and H16/1A^T^ as representing two novel species is supported by digital DNA–DNA hybridization (dDDH) values calculated with the Type (Strain) Genome Server using the recommended settings of the Genome-to-Genome Distance Calculator (GGDC) 2.1 [12] and by ANI values calculated by blast (ANIb) [13], MUMmer (ANIm) [13] and the tetranucleotide signature correlation index [13] (Table 1). Analysis of the latter three parameters was performed using JSpeciesWS [14]. With one exception, the values in each case were less than the threshold which is used to circumscribe strains as belonging to the same species [15] (Table 1). The one exception which did not fulfill the criteria for differentiating bacterial species was the tetranucleotide signature correlation index value of 99.7 % calculated in the comparison of *S. caledonicus* H8/1^T^ with *S. devriesei* CCUG 58238^T^. Divergence between ANI and tetranucleotide signature correlation index has been described previously with a possible explanation being that evolutionary or environmental forces may impede modifications in this genome signature despite genetic drift occurring [13]. Furthermore, the utility of the tetranucleotide signature correlation index and its correlation with dDDH and ANI in circumscribing or differentiating among staphylococci has received little attention to date. A further genome-based phylogeny of type strains of species of the genus *Staphylococcus* was generated using CSI Phylogeny 1.4 [16] applying the default setting as follows: minimum depth at SNP positions: 10×; minimum relative depth at SNP positions: 10 %, minimum distance between SNPs (prune): 10 bp; minimum SNP quality: 30; minimum read mapping quality: 25 and minimum Z-score: 1.96. Using *Staphylococcus aureus* DSM 20231^T^ as the reference genome, this analysis produced a single-nucleotide polymorphism tree comprising 19637 nucleotide positions (Fig. 3). In agreement with the GGDC-based *Staphylococcus* phylogeny, the results of this analysis indicate that H8/1^T^ is most closely related to, but distinct from, *Staphylococcus devriesei*; with H16/1A^T^ most closely related to, but distinct from, *Staphylococcus felis*. Before the advent of accessible whole-genome sequencing, the partial sequences of various housekeeping genes such as *dnaJ* [17], *tuf* [18], *sodA* [19] and *rpoB* [20] had been proposed to discriminant staphylococcal species. Whereas each of these single-gene approaches could distinguish H16/1A^T^ from *S. felis* DSM 7377^T^, none of them could be used to separate H8/1^T^ from *S. devriesei* CCUG 58238^T^, Table 1. The draft genome of H8/1^T^ is 2 503 367 bases in length with a DNA G+C content of 33.6 mol%, while the draft genome of H16/1A^T^ is 2 229 149 bases in length with a DNA G+C content of 34.8 mol%. These values are similar to those of their nearest relatives and consistent with the average and range values of the rest of the members of the genus *Staphylococcus* (Table 2).

**Fig. 1.**
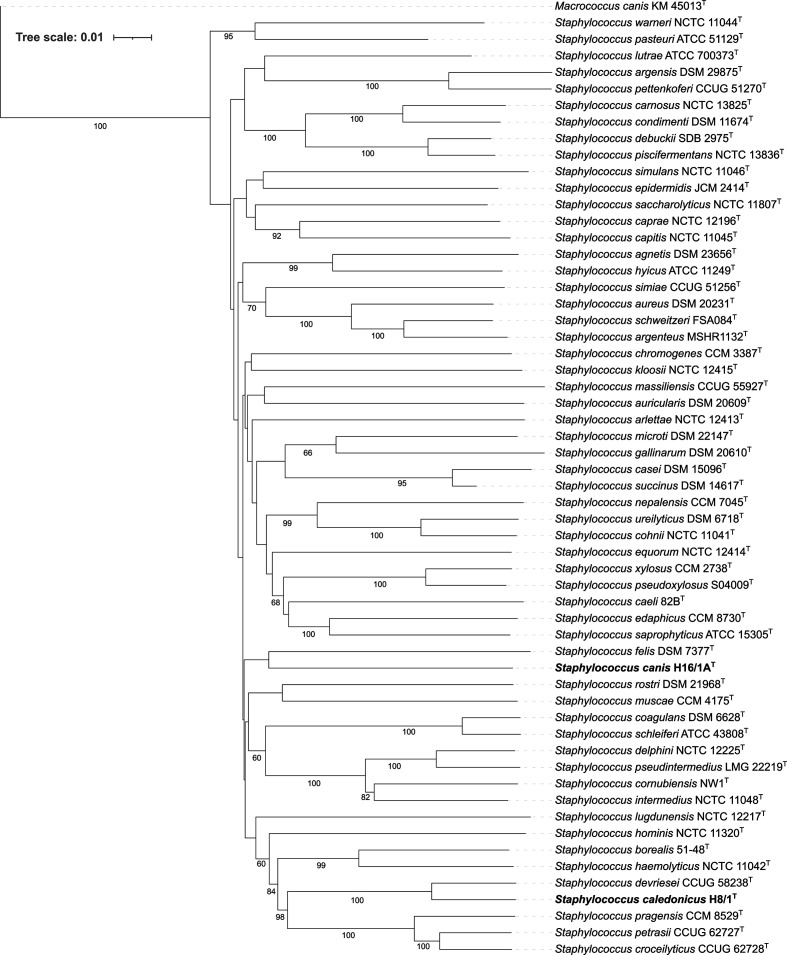
Balanced minimum-evolution tree containing *Staphylococcus caledonicus* H8/1^T^ and *Staphylococcus canis* H16/1A^T^ (highlighted in bold type) and all other type strains of members of the genus *Staphylococcus*. Tree generated using the Type (Strain) Genome Server (TYGS) (https://tygs.dsmz.de) [7] and inferred with FastME 2.1.6.1 [22] from GBDP distances calculated from genome sequences. The branch lengths are scaled in terms of GBDP distance formula *d*_5_ with pseudo-bootstrap support values shown of >50 % from 100 replications. *Mcrococcus canis* KM45013^T^ was included as the outgroup to root the tree. Accession numbers for all genomes in the analysis are provided in Table S1 (available with the online version of this article).

**Fig. 2.**
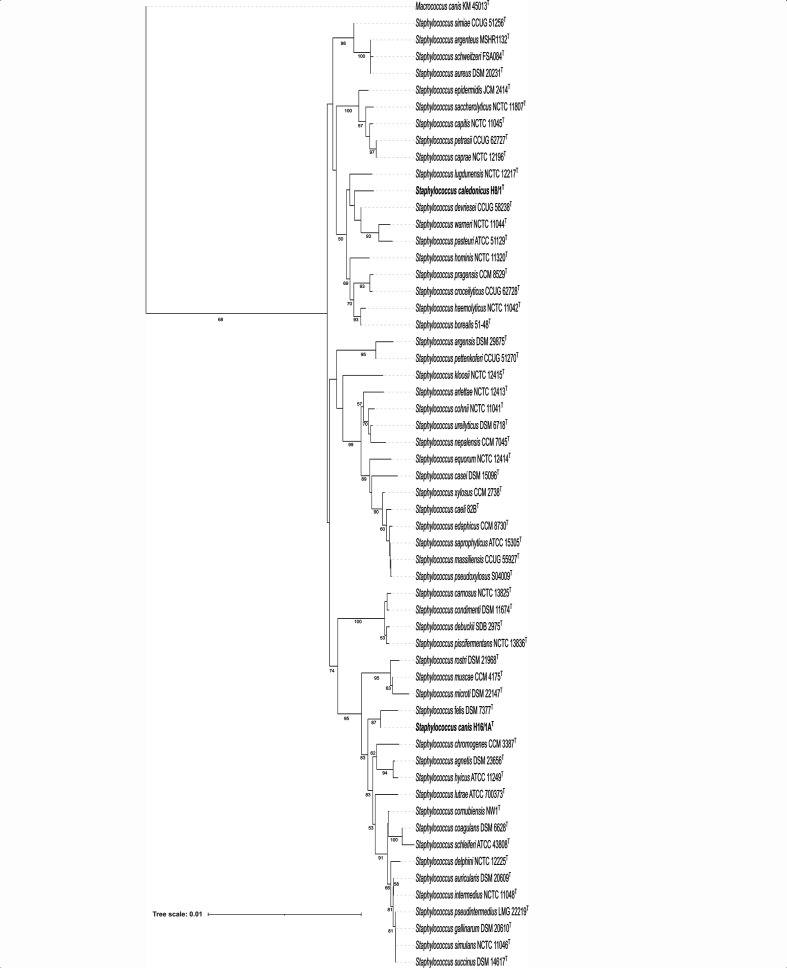
16S rRNA gene phylogeny of all species of the genus *Staphylococcus*. Tree generated using the Type (Strain) Genome Server (TYGS) (https://tygs.dsmz.de) [7] and inferred with FastME 2.1.6.1 [22] from GBDP distances calculated from 16S rRNA gene sequences. *Staphylococcus caledonicus* H8/1^T^ and *Staphylococcus canis* H16/1A^T^ are highlighted in bold type. The branch lengths are scaled in terms of GBDP distance formula *d_5_*. The numbers below branches are GBDP pseudo-bootstrap support values >50 % from 100 replications. *Macrococcus canis* KM45013^T^ was included as the outgroup to root the tree. Accession numbers for all isolates in the analysis are provided in Table S1.

**Fig. 3.**
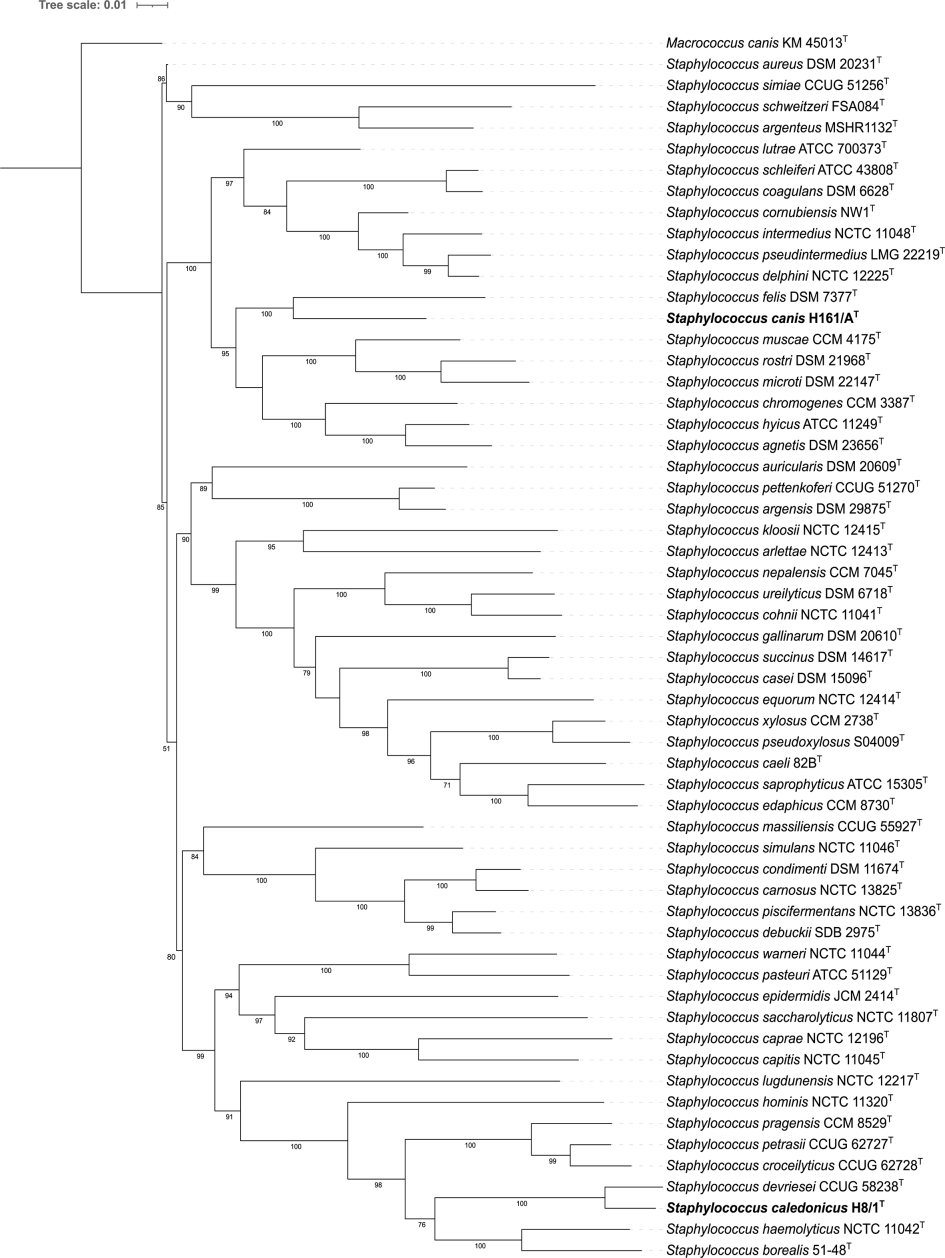
Maximum likelihood phylogeny of the genus *Staphylococcus* based on single-nucleotide polymorphisms. Tree generated using CSI Phylogeny 1.4 [16] with *S. aureus* DSM 20231^T^ as the reference genome with *M. canis* KM45013^T^ included as the outgroup to root the tree. *Staphylococcus caledonicus* H8/1^T^ and *Staphylococcus canis* H16/1A^T^ are highlighted in bold type. The analysis comprised 19637 nucleotide positions. Bootstrap values of >50 % are shown. Accession numbers for all isolates in the analysis are provided in Table S1.

**Table 1.** Genome and gene-based comparisons of *S*. *caledonicus* H8/1^T^ and *S*. *canis* H16/1A^T^ with their nearest-related species

Strain comparison			Gene/method (thresholds for circumscribing strains as the same species shown in brackets)						
	dDDH (>70 %) [12]	ANIb (>95–96 %) [13]	ANIm (>95–96 %) [13]	Tetra* (>99 %) [13]	16S rRNA (>98.7 %) [23]	*dnaJ* (>88.8 %) [17]	*tuf* (>98 %) [18]	*sodA* (>97 %) [19]	*rpoB* (>93.6 %) [20]
*S*. *caledonicus* H8/1^T^ compared with *S*. *devriesei* DSM 25293^T^	50.6 %	92.7 %	93.2 %	99.7 %	99.8 %	95.0 %	99.1 %	97.8 %	97.3 %
*S*. *canis* H16/1A^T^ compared with *S*. *felis* DSM 7377^T^	22.0 %	77.3 %	84.5 %	95.0 %	99.7 %	84.9 %	95.0 %	84.4 %	87.5 %

Accession numbers of analysed sequences: *S. devriesei* DSM 25293^T^; genome (for dDDH, ANI and tetranucleotide signature correlation index), GCF_002902625; 16S rRNA, UHCZ01000002; *dnaJ*, JX174277; *tuf*, FJ389248; *sodA*, MF679044; *rpoB* FJ389232. *S. felis* DSM 7377^T^ genome (for dDDH, ANI and tetranucleotide signature correlation index), GCA_002902185; 16S rRNA, D83364; *dnaJ*, AB234071; *tuf*, HM352941; *sodA*, AJ343908; *rpoB* AF325878.

* tetranucleotide signature correlation index.

**Table 2.** Genome lengths and DNA G+C contents of *S. caledonicus* H8/1^T^ and *S. canis* H16/1A^T^ in comparison to the most closely-related type strains of species of the genus *Staphylococcus* and the average for the genus *Staphylococcus*

Species/Genus	Genome length (bp)	DNA G+C content (mol%)
*S*. *caledonicus* H8/1^T^	2 503 367	33.6
*S*. *devriesei* CCUG 58238^T^	2 379 863	33.3
*S*. *canis* H16/1A^T^	2 229 149	34.8
*S. felis* DSM 7377^T^	2 408 386	35.1
*Staphylococcus* genus average* (range in brackets)	2 568 275 (204819–3171720)	34.5 (31.4–38.9)

*see Table S1 for strains, accession numbers and genome data used here, genus average of 55 species excluding *S. caledonicus* H8/1^T^ and *S. canis* H16/1A^T^.

Phenotypic characterisation of H8/1^T^ and H16/1A^T^ was performed using the API Staph system (bioMérieux) according to the manufacturer’s instructions alongside the type strains of *S. devriesei* DSM 25293^T^ and *S. felis* DSM 7377^T^ (Table 3). H8/1^T^ is distinguished from the related *S. devriesei* by the inability of the former to ferment lactose while the lack of arginine dihydrolase activity differentiates H16/1A^T^ from the related *S. felis*, (Table 3). Additionally, H8/1^T^ and H16/1A^T^ were tested for clumping factor and coagulase activity using rabbit plasma (with EDTA) and for DNAse activity using DNAse agar (Oxoid). In each case, both H8/1^T^ and H16/1A^T^ tested negative for these activities.

**Table 3.** Phenotypic characterisation of *S. caledonicus* H8/1^T^, *S. canis* H16/1A^T^ and closely-related type strains of species of the genus *Staphylococcus* Phenotype data generated in this study. Strains: 1. *S. caledonicus* H8/1^T^; 2. *S. devriesei* DSM 25293^T^; 3. *S. canis* H16/1A^T^; 4. *S. felis* DSM 7377^T^. +, Positive; −, negative; w, weak. Results presented from four independent replicates. Results presented in brackets represent those of the species as a whole, where such data are available, with symbols indicating: +, 90 % or more of strains are positive; −, 90 % or more of strains are negative; d, 11–89 % of strains are positive; nd, not determined. In the case of species-wide phenotypic data these are taken from original species descriptions [24, 25], except for fructose for *S. felis*, which was taken from [26].

Biochemical test	1	2	3	4
Acid production from:				
d-glucose	+	+	+	+
d-fructose	+	+	+	+[+]
d-mannose	−	−[d]	+	+[+]
maltose	+	+	+	−[−]
lactose	−	+	+	+[+]
trehalose	+	+	+	+[+]
d-mannitol	+	+[d]	+	+[d]
xylitol	−	−	−	−[−]
melibiose	−	−	−	−
raffinose	−	−	−	−[−]
d-xylose	−	−	−	−[−]
sucrose	+	w	−	−[d]
methyl α-d-glucopyranoside	−	−	−	−
n-acetylglucosamine	−	−	w	w
reduction of nitrates to nitrites	+	w[d]	+	+[+]
alkaline phosphatase	w	−	+	+[+]
Voges–Proskauer	w	+	w	+
arginine dihydrolase	+	+[+]	−	+[+]
urease	w	+[d]	+	+[+]

Antimicrobial sensitivity testing was performed using the Vitek2 system (bioMérieux) according to the manufacturer’s instructions. Using the AST-GP80 card and applying the CLSI 2017 interpretations for coagulase-negative staphylococci, both H8/1^T^ and H16/1A^T^ were susceptible to all the antimicrobials tested, which were as follows: amoxicillin/clavulanic acid, benzylpenicillin, cefovecin, cefoxitin (screen), ceftiofur, chloramphenicol, clindamycin, doxycycline, enrofloxacin, erythromycin, gentamicin, inducible clindamycin resistance, kanamycin, marbofloxacin, neomycin, nitrofurantoin, oxacillin, pradofloxacin, tetracycline and trimethoprim/sulfamethoxazole. No known antimicrobial resistance genes (perfect and strict hits) were identified in either H8/1^T^ and H16/1A^T^ on using The Comprehensive Antibiotic Resistance Database (CARD) (https://card.mcmaster.ca/) [21].

## Description of *Staphylococcus caledonicus* sp. nov.

*Staphylococcus caledonicus* (ca.le.do′ni.cus. L. masc. adj. *caledonicus*, from Caledonia (Scotland), the country where the type strain was isolated).

Gram-stain-positive, non-spore forming, facultative anaerobe, forms non-pigmented, smooth, circular colonies about 1–2 mm in diameter with entire margins on Columbia horse blood agar after 18 h incubation at 37 °C. Able to produce acid from d-glucose, d-fructose, maltose, trehalose, d-mannitol and sucrose but not from d-mannose, lactose, xylitol, melibiose, raffinose, d-xylose, methyl α-d-glucopyranoside or *N*-acetylglucosamine. Has arginine dihydrolase activity and is able to reduce nitrates to nitrites. Catalase-positive and negative for clumping factor, coagulase and DNAse.

The type strain of *S. caledonicus*, H8/1^T^ (=NCTC 14452 ^T^=CCUG 74789^T^), was isolated from a healthy dog in Scotland during 2018. The draft genome of H8/1^T^ is 2 503 367 bases in length with a DNA G+C content of 33.6 mol%, it comprises 38 contigs with an average coverage of approximately 85-fold. The genome sequence data from H8/1^T^ is available under these accession numbers: BioSample, SAMN15065541; Sequence Read Archive, SRR11909362; and assembly, JABTXV000000000.

## Description of *Staphylococcus canis* sp. nov.

*Staphylococcus canis* (ca′nis L. gen. masc./fem. n. *canis*, of a dog, in reference to the host from which the type strain was isolated).

Gram-stain-positive, non-spore forming, facultative anaerobe, forms non-pigmented, smooth, circular colonies about 1–2 mm in diameter with entire margins on Columbia horse blood agar after 18 h incubation at 37 °C. Able to produce acid from d-glucose, d-fructose, d-mannose, maltose, lactose, trehalose and d-mannitol but not from xylitol, melibiose, raffinose, d-xylose, sucrose or methyl α-d-glucopyranoside. Has alkaline phosphatase and urease activity and is able to reduce nitrates to nitrites. Catalase-positive and negative for clumping factor, coagulase and DNAse.

The type strain of *Staphylococcus canis*, H16/1A^T^ (=NCTC 14451^T^=CCUG 74790^T^), was isolated from a healthy dog in Scotland during 2018. The draft genome of H16/1A^T^ is 2 229 149 bases in length with a DNA G+C content of 34.8 mol%, it comprises 143 contigs with an average coverage of approximately 92-fold. The genome sequence data from *Staphylococcus canis* H16/1A^T^ is available under these accession numbers: BioSample, SAMN14548534; Sequence Read Archive, SRR11498036; and assembly, JABANU000000000.

## Supplementary Data

Supplementary material 1
